# Gut microbiota: an ideal biomarker and intervention strategy for aging

**DOI:** 10.20517/mrr.2023.68

**Published:** 2024-01-01

**Authors:** Xuan Xu, Tangchang Xu, Jing Wei, Tingtao Chen

**Affiliations:** ^1^Institution of Translational Medicine, Jiangxi Medical College, Nanchang University, Nanchang 330031, Jiangxi, China.; ^2^Huankui Academy, Jiangxi Medical College, Nanchang University, Nanchang 330031, Jiangxi, China.; ^#^Authors contributed equally.

**Keywords:** Aging, gut microbiota, microbial biomarker, anti-aging intervention

## Abstract

Population aging is a substantial challenge for the global sanitation framework. Unhealthy aging tends to be accompanied by chronic diseases such as cardiovascular disease, diabetes, and cancer, which undermine the welfare of the elderly. Based on the fact that aging is inevitable but retarding aging is attainable, flexible aging characterization and efficient anti-aging become imperative for healthy aging. The gut microbiome, as the most dynamic component interacting with the organism, can affect the aging process through its own structure and metabolites, thus holding the potential to become both an ideal aging-related biomarker and an intervention strategy. This review summarizes the value of applying gut microbiota as aging-related microbial biomarkers in diagnosing aging state and monitoring the effect of anti-aging interventions, ultimately pointing to the future prospects of microbial intervention strategies in maintaining healthy aging.

## INTRODUCTION

Aging is a physiological process that individuals must undergo to death. The World Health Organization (WHO) defines it as “the gradual accumulation of molecular and cellular damage over time”^[1]^, which further involves all tissues and triggers systemic structural breakdown and functional decline. As estimated, people aged 65 or older accounted for 9.1% of the world’s overall population in 2019, imposing a heavy burden on the national economy and healthcare system^[2]^. Glycation, oxidation, inflammation, and dysbiosis have been emphasized for a prominent correlation with aging^[3]^, which benefits both the elaboration of aging status and the expansion of the anti-aging drug spectrum. With the deepening of research these years, the position of gut microbes in aging events has been progressively better described.

Trillions of microbes constitute the broadest human symbiotic microecology, co-operating in the maintenance of the body’s immune defense^[4]^, nutrient absorption^[5]^, and neuroprotection^[6]^. Technical advancements in high-throughput sequencing and multi-omics analysis have facilitated the study of microbiota in individual growth and metabolism, and the role of gut microbes in host aging has been increasingly elucidated^[7]^. Metagenomic sequencing of fecal samples from multiple longevity families implicated that the diversity of gut microbiota and the abundance of bacterial metabolites in aging populations were lower than in younger generations^[8]^. However, specific adjuncts such as fecal microbiota transplantation (FMT)^[9]^ or probiotic supplementation^[10]^ could, in turn, help retard aging, or at least enhance the survival quality of the elderly. At this scale, gut microbes can be interpreted as a potential aging-related biomarker and, through microbiota modulation, hold promise for healthy aging or even aging reversal.

This mini-review systematically depicts the role of gut microbiota in distinguishing different aging patterns, and its feasibility in retarding aging or maintaining healthy aging, thus outlining a blueprint for the application of gut microbes as biomarkers or intervention targets in active health.

## BIOLOGICAL OR CLINICAL PERSPECTIVES TO DEFINE AND CHARACTERIZE DIFFERENT PATTERNS OF AGING

### The definition and signatures of aging

Aging can be interpreted as a state of generalized degeneration. The superimposition of genetic and environmental factors contributes to the complexity of aging, and the intuitive evaluation of clinical aging by chronological age is no longer applicable. The inconsistency between physical function and age has become highly pronounced, making biological age a better criterion. Cellular senescence, the initiating link of systemic aging, first triggers molecular damage, which in turn induces oxidative stress, inflammatory immunity, nutritional dysregulation, metabolic impairment, and neurodegeneration in the organism, and eventually presents signs of aging^[11]^. On the basis of biological alterations, aging can be classified into physiological and pathological aging^[12]^. The latter is often associated with diabetes^[13]^, cardiovascular diseases^[14]^, cancers^[15]^, and neuro-degenerative diseases^[16]^, resulting in poor life quality and, ultimately, a shortened lifespan.

It is worth emphasizing that although aging is inevitable, the pursuit of delayed aging or at least healthy aging is attainable with flexible monitoring and potent interventions. The identification and detection of aging-related biomarkers are paramount for quantifying the level of physical function and evaluating the effect of behavioral factors. The ideal aging-related biomarker, as defined by the American Consortium for Aging Research (AFAR), should enable individualized marking and systematic monitoring of aging, as well as longitudinal non-invasive follow-up in subjects^[17]^. The DNAmAge, to determine chronological age based on the methylation of deoxyribonucleic acids, and GlycanAge, to track the effects of lifestyle interventions on aging based on the glycosyl molecules attached to immunoglobulin G, are among the more established and commercially available biomarkers of aging^[18]^. Although none have yet been approved for clinical application, their accuracy in marking aging is well accepted. Other studies have also focused on the host level of age-related biomarkers; it is possible that the combination of serum biomarkers such as inflammatory bodies^[19]^ (indicating inflammatory aging), Sirtuins^[20]^ (associated with malnutrition risk and frailty phenotype), and Clusterin^[21]^ (monitoring neurodegeneration) will be adopted in conjunction for the quantification of aging. A common shortcoming, however, is the unsatisfactory efficacy of the above indicators in characterizing inter-individual variabilities and environmental sensitivities.

Gut microbiota can be summarized as bacterial communities that colonize or temporarily reside in the gastrointestinal cavity^[22]^. The spatiotemporal variations in the abundance, composition, and vitality of microbiota constitute a vast microecology, thus bridging the human body and the external environment, and exerting a pivotal impact on health and disease. The key point lies in the role of the gut microbiome in characterizing diseases such as gastrointestinal cancers, diabetes, and obesity. For instance, significant correlations have been reported between *Helicobacter pylori* infection and the high risk of gastric lymphoma^[23]^, as well as the indication of *Fusobacterium nucleatum* abundance in the prognosis of colorectal cancer^[24]^. These years, with the inclusion of dysbiosis in aging signatures in 2023^[12]^ and multiple researches elucidating the function of microbiota interventions in aging-related diseases, here we reiterate the possibility of using gut microbiota as biomarkers to predict aging and evaluate the efficacy of anti-aging interventions.

### Gut microbiota as biomarkers to diagnose and monitor aging

#### Gut microbiota as predictive biomarkers

Multiple large-sample, multi-center, and group-based studies have partially clarified the inter- and intra-population differences in aging-related microorganisms. Metagenomic sequencing of gut microbes from 32 long-lived families across three generations (including centenarians, the elderly, and the younger) revealed reduced gut microbiota diversity and functional deficits of associated essential amino acids in the elderly. However, interestingly, a significant enrichment of anti-inflammatory bacteria such as *Desulfovibrio piger*, *Gordonibacter pamelaeae*, and *Ruminococcaceae bacterium* D5 was observed in centenarians compared to younger and older age groups, and short-chain fatty acid high-yielding bacteria like *Akkermansia muciniphila* and other potential probiotic behave similarly, which may contribute to the health maintenance of aging individuals^[25]^. In addition, an observational study of four age groups, young, old, centenarians, and semi-centenarians, proposed the concept of enterotype^[26]^, which divides the gut microbiota into multiple functional core microbiome clusters. One group of enterotypes containing Bacillariophyceae, *Prevotella*, and Rumenococcaceae decreased in abundance in the advanced age groups, suggesting that the delineation and definition of enterotypes may help describe the downgrading of overall functional levels in the aging population and also serve as potential aging-related microbial biomarkers.

Further, healthy elderly individuals also exhibit an evident flora profile compared to the non-healthy ones. *Akkermansia* spp. and *Erysipelaceae* UGG were the units with significantly increased abundance in the microbial taxa of a healthy aging population^[27]^. Monitoring data on immune function, blood pressure, and body weight in both groups also illustrated the role of quantitative changes in taxa such as *Ruminococcus*, *Prevotella*, *Oscellobacter* CAG, and *Bacteroides* in the gut microbiome in healthy aging^[28]^. Notably, a study has confirmed the feasibility of using altered *Sartorius* spp./*Pasteurella* ratios as an essential microbial alteration for aging-associated sarcopenia; the role of gut bacteria-derived metabolites in characterizing aging-induced neurodegeneration has also been highlighted in another study^[29]^, which inspired us to construct a subsystem of aging-related diseases with associated microbial factors for a more precise aging representation.

#### Gut microbiota as reactive biomarkers

Response to interventions is another major criterion for screening aging-related biomarkers. Utilizing diet and drug interventions as the primary means of anti-aging has become a consensus, in which gut microbial alterations as reactive biomarkers make sense. Modification of dietary strategies such as caloric restriction (CR)^[30]^ and intermittent fasting (IF)^[31]^ has been proven to maintain health and prolong lifespan in a target of rapamycin (TOR) signaling pathway-dependent or -independent manner, and this is, at least partially due to gut improvement. Therefore, the detection of α-diversity and β-diversity, which indicate microbiome abundance and diversity, can well reflect the intervention effect^[32]^. Furthermore, biased diets fortified with substances such as phenols and fiber intake have been observed to exhibit dual benefits of delaying aging and regulating the gut microbiota. The above effect can be signified by the enhanced abundance of probiotics such as *Bifidobacterium* and *Lactobacillus*^[33]^. In addition, medications contribute to the anti-aging campaign. TOR signaling inhibitor Rapamycin^[34]^, chronic disease targeting drug Resveratrol^[35]^, and the Chinese pharmaceutical preparation FuFang Zhenshu TiaoZhi (FTZ) have all been adopted in the preventive healthcare of the elderly. Unexpectedly, gut microbial changes can also describe drug efficacy. The FTZ-administered aging mice model showed an extended lifespan, along with a decline in the level of *Clostridium erysipelae*, and the raised abundance of probiotics such as *A. muciniphila* and *Clostridium butyricum* existed at the same time^[36]^. However, the problem is that the causal association between the anti-aging capacity of the above interventions and the altered microbiota remains unknown.

## THE INVOLVEMENT OF GUT MICROBIOTA IN HEALTHY AGING

### Inflammatory immunity

The capacity of gut microbiota to mark aging is inextricably linked to the biological mechanisms by which it acts on the body, dominated by immunomodulation. Intestinal stem cells (ISCs), located in the crypts, serve as the cellular basis for intestinal epithelial renewal and mucosal permeability^[37]^. Gut microbes in healthy adults can interact with ISCs through their bacterial components and metabolites, on the one hand activating the dual oxidase to generate reactive oxygen (ROX) in response to bacterial uracil^[38]^, and on the other hand initiating the immune deficiency (IMD)/Relish pathway to enhance the expression of antimicrobial peptides (AMP)^[39]^, which together mediate the maintenance of the balance between gut and microbiome, as well as normal immune state. The intestinal mucus layer also depends on the commensal microbiota to induce the release of secretory immunoglobulin A and inflammatory factors such as interleukin-6, interleukin-10, and tumor necrosis factor-α, thereby achieving immune suppression and killing of opportunistic pathogens^[40]^.

Due to the hyperfunction of pro-inflammatory T-cell populations (e.g., TH1 and TH17 cells), defects in immune surveillance, and loss of self-tolerance, the intrinsic immunity of the elderly is susceptible^[41]^. In this case, unhealthy aging accompanied by intestinal dysbiosis directly disrupts the delicate balance that exists in the gut of healthy individuals, as described above, which may at least partially explain the onset of systemic low-grade inflammation. Specifically, the upregulated expressions of ROX and AMP should be blamed. ROX is a key molecule mediating cellular senescence, while sustained ROX production also leaves the intestinal epithelium in a state of chronic oxidative stress damage^[38]^. Impaired intestinal barrier integrity and elevated mucosal permeability not only hinder the digestion and absorption of nutrients in the elderly, but also accelerate the leakage of pathogens and harmful substances into the circulation, raising the incidence of aging-related diseases^[37]^. Studies have also confirmed that in *Drosophila*, the upregulation of AMP would make the peptidoglycan recognition protein-dependent gut development and longevity modulation difficult to achieve^[42]^. All of the above prompt that the imbalance of gut symbiotic microbiota-host interactions may induce a chain reaction of aging and short lifespan via activating conserved immune pathways. Therefore, surveillance and maintenance of normal intestinal microecology are required for healthy aging.

### Nutrient metabolism regulation

Nutrition is the main common factor that links both gut microbes and hosts; thus, the microbiome may influence lifespan and health by supplementing nutrition or regulating the body’s nutritional metabolic signaling pathways^[43]^. Cellulose, for instance, can be decomposed into short-chain fatty acids by *Ruminococcus* and *Butyrivibrio*, *etc.*, converting from being unavailable for intestinal absorption to indispensable bioactive molecules. These bacterial-derived metabolites have been shown to be effective in the amelioration of basic diseases in the elderly, such as type 2 diabetes and obesity^[44]^. Insulin/insulin-like growth factor 1 (IGF-1) signaling (IIS), the first signaling proven to be involved in aging regulation, was found to be somewhat relevant to the nutrient metabolism of gut bacteria^[45]^. *Caenorhabditis elegans* model with healthy bacterial composition features proper intestinal nutrient supply and sensory capacity, and the IIS pathway is sustained in low activation, thus mitigating the host aging^[46]^. Similar situations also occur on other nutritional metabolic pathways associated with the IIS, such as Sirtuins, Forkhead Box O (FOXO)^[47]^, and mammalian TOR pathways^[48]^. For instance, *Δhns Escherichia coli* administration could extend the lifespan of *C. elegans* by activating decay accelerating factor-16 (DAF-16)/FOXO family transcription factors, and this effect is independent of the IIS pathway^[49]^.

### Neuroprotection

Neurodegeneration is the typical feature of individual aging in the central nervous system, mainly manifested as motor and cognitive decline, for which the onset of Parkinson’s disease^[50]^ and Alzheimer’s disease^[51]^ is most common. For the gut-brain axis, a figurative perception defines it as the collection of signaling pathways between the central and enteric nervous systems that enable a bidirectional connection between the gut and the brain through immune, metabolic, and neuroendocrine mediators^[52]^. Studies have shown that healthy gut microbiota could prevent Alzheimer’s disease through the gut-brain axis, an effect that relies on the interaction and signaling of microecology-associated neurological and humoral factors. In particular, the restriction of trimethylamine oxide levels inhibits β-amyloid formation, neuroinflammation, and tau phosphorylation, mitigating the further progression of mild cognitive impairment to organic lesions in older individuals^[53]^. In addition, metabolites of gut microbes such as secondary bile acids and tryptophan derivatives can modulate the bile acid profile, serotonin synthesis pathway, and the number of brain-derived neurotrophic factors, which, in turn, yield positive guidance for the learning behavior, memory recognition, and emotional expression in the organism^[54,55]^.

## INTERVENTIONS FOR HEALTHY AGING: GUT MICROBIOTA TARGETED

### Fecal microbiota transplantation

The mechanism of gut microbiota-host interaction provides another perspective on anti-aging research, that is, in addition to marking aging, gut microbes themselves or strategies targeting gut microbes possess the potential for anti-aging effects. FMT transplants functional microbiota from the feces of healthy donors into the gut of patients, treating both intestinal and extra-intestinal diseases through microecological remodeling. Initially, FMT was tentatively applied in *Clostridium difficile* infection, stubborn constipation, and intestinal immune deficiency, with demonstrable efficacy^[56]^. As standardized FMT takes hold, there is growing evidence to support its anti-aging effect. In a premature aging mice model, the experimental group transplanted with fecal bacteria from wild-type mice showed a 13.5% prolonged average lifespan and a 9% enhanced maximum survival rate^[57]^. After transplantation of fecal bacteria from long-lived elders into mice, alleviation of aging indicators and improved richness of *Lactobacillus* spp. and short-chain fatty acid-producing bacteria were observed compared to controls^[58]^. Another study then illustrated that FMT improved aging-related signatures in the eye, brain, and gut by repairing intestinal barrier, reversing systemic inflammation, and refining nutrient metabolism^[59]^.

In addition, the advantage of FMT also lies in the capacity to treat various aging-related diseases through the distal effects of axial networks such as the gut-brain axis, gut-skin axis, and gut-muscle axis. Enterobacteria transplantation from young mice into premature aging mice revealed that the rejuvenated microbiota could reverse aging-related dementia and cognitive decline to some extent via hippocampal restoration^[60]^. In a trial that involved 110 middle and old-aged women with dry, wrinkled skin, a 21.73% improvement in skin elasticity and evident reduction in wrinkle depth were observed after consistently administrating a specially formulated microbiota drug for 12 weeks^[61]^. The senile osteoporosis cohort also presented significantly better histologic indices, such as bone volume and trabecular bone thickness, than the controls after receiving FMT^[62]^. The above experimental findings could be considered compelling arguments for FMT retarding aging and alleviating aging-related lesions. Remarkably, metabolomic analysis showed a gradual resumption of the originally dysregulated bile acid profile after FMT, which elucidates the contribution of gut microbiota to the endocrine metabolism of the organism^[63]^.

Along with the benefits, we must also be alert to the shortcomings of FMT at this stage. The occurrence of adverse events is a concern, since cases of death from resistant bacterial infections after FMT have been reported, as well as ineffective transplantation, immune rejection, and gastrointestinal side effects^[64]^. In addition, FMT in unhealthy aging populations is intended to mitigate or even reverse the negative effects of aging, but follow-up observational data on whether offsetting these effects on a large scale is feasible in the long term is lacking. The specificity of the elderly also requires more rigorous process management for the anti-aging application of FMT compared to current regulatory guidelines, including ethical approval, metrics assessment, and adverse event monitoring.

### Probiotics, prebiotics and postbiotics

WHO defines probiotic living microorganisms as those that benefit the health of the host when supplemented adequately^[65]^. Specific probiotics maintain health and exert aging-delaying effects in multiple dimensions, such as dysbiosis regulation, anti-inflammation, and neuroprotection. In addition to the well-known probiotics with anti-aging properties, such as *Lactobacillus salivarius*, *Lactobacillus galacteri*, and *Lactococcus lactis*, other probiotics that have been observed to extend lifespan are emerging these years^[66]^. Recent studies have revealed that *Lactobacillus plantarum* JBC5 administration prolonged the average longevity of Drosophila by 27.81%, mainly through the modulation of the serotonin signaling pathway^[67]^. *Bifidobacterium adolescentis* could raise superoxide dismutase values in serum and alleviate neuropathies by combating oxidative stress^[68]^. *Lacticaseibacillus rhamnosus* Probio-M9 significantly extended nematode lifespan with a calorie-independent DAF-2 pathway and P38 signaling cascade^[69]^. Moreover, *Clostridium pratense* (the main producer of butyrate) and *Bacillus* spp. (production of glycosyl hydrolases) also deliver superior anti-aging properties by regulating the balance of small molecules, such as short-chain fatty acids in the gut^[70]^. Probiotics from different phyla work through different biological pathways for anti-aging; judging which is superior or inferior makes little sense. Currently, *A. muciniphila* is a well-deserved hotspot with definite anti-glycemic and anti-aging performance^[71]^. After oral administration of *A. muciniphila*, the biosynthesis of secondary bile acids in premature aging mice was also enriched, thereby exerting the protective effect on accelerated aging^[72]^. Since the relevant research is just starting, the industrialization of *A. muciniphila* in anti-aging still has a long way to go. The results of microbiota localization also remind us that the action site of supplemented probiotics is rather limited and the species is relatively homogeneous, making it difficult to achieve the holistic regulation of the gut microecology.

Except for probiotics themselves, prebiotics such as inulin, oligosaccharides, and polyphenols have been repeatedly shown to selectively promote the growth of beneficial intestinal bacteria without being digested and absorbed by the host, thus providing a certain anti-aging effect. In particular, oligosaccharides have been proven in previous studies to regulate gut homeostasis by activating *Bifidobacteria* and inhibiting the production of intestinal spoilage substances^[73]^. Recent studies have observed the proliferation of *A. muciniphila* in the gut after consumption of oligosaccharides, which may also be one of the anti-aging mechanisms of prebiotics^[74]^. The processed probiotic metabolites, collectively known as postbiotics, contain various active ingredients such as vitamins, proteins, and SCFAs^[75]^. Studies have reported the superior probiotic and anti-aging properties of postbiotics in the internal environment compared to living microbes; thus, preliminary progress has been made in the application of postbiotic extracts in skin aging and certain chronic diseases^[76]^. The intake of prebiotics and postbiotics is undoubtedly safer compared to direct supplementation with exogenous probiotics, but the extent to which their actual anti-aging efficacy can be attributed to themselves is worth speculating.

### Dietary intervention

Compared to supplements, interventions based on a holistic diet may better reset the gut microbiota and thus achieve long-term health maintenance for the aging population. CR that does not induce malnutrition is optimal for extending lifespan. A study concluded that a fasting strategy of cutting food intake by 30% for two weeks shaped the *Lactobacillus*-dominant gut microbiota in mice, and the median lifespan of the experimental group increased by 20.8% after further full-life-cycle dieting^[77]^. As estimated, the standard CR adopted in most studies could extend the lifespan by an average of 10%-20%^[78,79]^. The other is the Mediterranean diet, which emphasizes the intake of fruits, vegetables, and grains, and limits the consumption of red meat and saturated fats. The maintenance of health-related microbiome, including enrichment of potential probiotics such as *Enterococcus faecalis* and *Rhodobacter sphaeroides* and depletion of deleterious bacteria such as *Rhodobacter torques* and *Collinsella*, has been found in the elderly with Mediterranean diet intervention^[80]^. This contributes to lowering inflammatory levels, improving cognitive function, and reducing frailty, implying a healthier aging condition. The accessibility and health benefits of the Mediterranean diet make it a recommended preventive medicine therapy, but its relevance for generalization in anti-aging practice still requires individualized assessment by clinicians.

## CONCLUSION

Aging is the joint outcome of both genetic and environmental factors. Microorganisms have been observed to be involved in multiple aging-related diseases, and their inter-individual variability renders them an alternative as aging-related markers and intervention targets. In this review, underlying mechanisms of microbiota on host aging were explored, including inflammatory immunity, nutrient metabolic regulation, and neuroprotection. By summarizing the microbial features of aging populations, the value of various bacteria such as *A. muciniphila* and derived metabolites as biomarkers in characterizing aging and monitoring anti-aging interventions was identified. Additionally, given the active interactions between gut microbiota and host, we generalized the current status and future prospects of microbiota-targeted anti-aging interventions such as FMT and probiotic supplementation, attempting to satisfy the healthy aging of the widest range of aging populations.

Setting gut microbiota as an entrance to explore aging characterization and anti-aging interventions is certainly a revolutionary mindset, but problems exist as well. The field of gut microbiota as aging-related biomarkers is still in its infancy, and the characterization of aging by microbiota remains only qualitative, lacking specific quantitative indicators to describe the correlation between both, thus necessitating the standardization of authoritative guidelines^[81]^. Secondly, the detailed mechanism between gut microbiota alterations and host aging is not yet clarified, and the actual microbial involvement in the aging process may be exaggerated or obscured. Thus, microbiota intervention combined with other anti-aging therapies might be a viable option. In addition, given the complexity of aging, model organisms such as nematodes, Drosophila, and mice^[82]^ are often employed to simplify variable control and experimental manipulation. This inevitably erases species-specific and inter-individual variations in human aging; thus, how to transfer the findings to humans remains to be investigated. Inserting primate models (e.g., rhesus monkey)^[83]^ as an intermediate transition may work. Certainly, the microbiota system is already highly sophisticated, and even more so with human experiments. Human anti-aging studies must be optimized with updated statistical tools and multi-omics analysis to obtain more reference data.

## DECLARATIONS

### Copyright

© The Author(s) 2024.
